# Balancing Risk and Reconstruction: A Comprehensive Review of Complications in Delayed Breast Reconstruction

**DOI:** 10.3390/jcm15124474

**Published:** 2026-06-09

**Authors:** Lamorna Coyle, Gabrielle Odoom, Ilexa Schechter, Neil Tanna, Joseph A. Ricci

**Affiliations:** 1 Donald and Barbara Zucker School of Medicine at Hofstra/Northwell, Hempstead, NY 11549, USA; lcoyle1@northwell.edu (L.C.); ischechter@northwell.edu (I.S.); 2 Department of Plastic and Reconstructive Surgery, Northwell Health, Queens, NY 11004, USA; gabbyodoom9@gmail.com (G.O.); ntanna@northwell.edu (N.T.)

**Keywords:** delayed breast reconstruction, delayed DIEP flap reconstruction, implant-based reconstruction, reconstruction after mastectomy

## Abstract

Breast cancer accounts for nearly one in four cancer diagnoses amongst women, with 36–50% of patients electing to undergo post-mastectomy breast reconstruction. Though immediate reconstruction has risen in popularity due to higher patient satisfaction scores, factors such as individual patient anatomy, patient preference, and adjuvant oncologic treatments may preclude this option for some patients. In such circumstances, a delayed approach to reconstruction offers a promising alternative, often offering comparable aesthetic results with lower rates of major complications. Autologous, implant-based, and hybrid reconstructive techniques may all be applied in a delayed setting at a time point clinically distinct from oncologic resection, with each technique carrying unique advantages and risks that must be evaluated in the context of patient-specific factors. By providing an overview of common complications associated with various delayed breast reconstruction modalities, this review seeks to synthesize the current approaches to prevention, management, and treatment of reconstructive obstacles and outcomes to foster shared decision-making, individualized surgical planning, and optimal reconstructive results.

## 1. Introduction

Breast cancer is the most common malignancy among women worldwide, accounting for nearly one in four cancer diagnoses in women with approximately 2.3 million new cases annually [1]. Projections estimate that by 2050, the global incidence of breast cancer will surpass 3.5 million new cases per year, while annual mortality is expected to rise by nearly 68% [2]. Current data estimates that between 36–50% of women undergo breast reconstruction following mastectomy—most commonly in the immediate setting—a trend primarily attributed to lower cost and greater afforded psychosocial well-being [3,4]. While less prevalent, the typical timeframe for delayed reconstruction falls within 6 to 12 months after mastectomy but may also occur years later, often after completion of adjuvant therapy. Mastectomy performed at the same time as reconstruction may produce a breast pocket inappropriately sized for a permanent implant, a product of extensive axillary dissection and resection that frequently extends beyond the breast footprint. Delaying the final breast reconstruction for several months after initial mastectomy therefore grants an opportunity for staging and creation an optimal breast pocket using tissue expanders. Through this technique, the mastectomy skin flap is allowed time to heal, with its final borders conforming to the size and position of the tissue expander. Although consensus suggests comparable aesthetic outcomes between immediate and delayed reconstruction, a staged approach may help alleviate cosmetic difficulties in certain patient populations. Several studies indicate that delayed reconstruction may also be associated with lower rates of major complications—including reconstructive failure—though evidence remains mixed [5,6,7,8]. A delayed approach may garner high consideration in patients requiring post-mastectomy radiation therapy (PMRT), those with significant medical comorbidities, inflammatory breast cancer, or patients who may benefit from additional time to make informed decisions regarding their reconstruction [5].

The need for PMRT is a primary indication for delayed breast reconstruction. PMRT is advised in patients with high-risk breast cancer features, including involvement of regional lymph nodes, primary tumors larger than 5 cm (T3–T4), or positive surgical margins that are not amenable to complete resection [9]. Pooled analyses have demonstrated significant reductions in locoregional recurrence, as well as improvements in disease-free and overall survival, among women with pT3–pT4 or node-positive breast cancer who received PMRT [9]. Reconstructive results in the setting of PMRT vary considerably and are critically dependent on reconstructive technique. Autologous flaps have been irradiated with variable success rates: while some studies note increased rates of poor wound healing, fat necrosis, fibrosis, and volume loss, others report minimal change, with literature generally supporting insignificant differences in overall complication rates between irradiated and non-irradiated autologous reconstructions [10,11,12]. By contrast, outcomes in the setting of implant-based reconstruction are disproportionately affected by PMRT [11]. While some data suggests that a two-stage approach is associated with greater rates of reconstructive failure, there is also data that supports a higher risk of capsular contracture among immediate direct-to-implant reconstruction recipients, a crucial determinant of poor cosmesis, pain and discomfort [10,11]. In general, autologous methods are preferred in the setting of PMRT, though patient-specific factors frequently drive surgical counseling. Recent trends towards hybrid intensity-modulated radiation therapy may reduce off-target damage to surrounding tissue, potentially offering a superior radiation method compared to traditional protocols and modifying the impact of PMRT on breast reconstruction results [13]. Current recommendations from the National Comprehensive Cancer Network state that optimal timing of delayed breast reconstruction is at least 6 months after the conclusion of radiation [12].

Comorbidity burden must also be carefully considered when determining the optimal timing of breast reconstruction. Concomitant diagnoses of poorly controlled diabetes, obesity, smoking, or inherited coagulopathy may account for up to 12% of delayed reconstructive decisions, as these factors are associated with higher rates of surgical complications such as infection, wound necrosis, and implant failure [14,15]. Women who choose to undergo breast reconstruction often carry a concurrent diagnosis of malignancy, increasing the risk of venous thromboembolism (VTE). Importantly, the incidence of VTE is higher among patients undergoing skin-sparing or nipple-sparing mastectomy with immediate breast reconstruction compared with patients who undergo delayed reconstructive procedures [16]. Other variables, such as age greater than 65, history of chemotherapy, obesity, and chronic lung disease, can further elevate VTE risk and threaten immediate reconstruction results [17]. Given these considerations, a delayed reconstructive approach may be warranted in patients with a high comorbidity burden.

Although some patients may benefit from additional time to accept their diagnosis and plan the course of their surgery, patients with mastectomy defects awaiting delayed reconstruction have exhibited greater interim distress and dissatisfaction with their breast appearance compared to patients who received immediate breast reconstruction, though higher baseline body image concerns in the delayed reconstruction group may account for some of this discrepancy [18]. Delayed reconstruction has been associated with worse short-term outcomes in body image (*p* = 0.01), sexuality (*p* = 0.01), psychological (*p* <0.01) and sexual well-being (*p* <0.01) compared to these metrics in immediate reconstruction counterparts [18]. However, both immediate and delayed groups demonstrate comparable long-term outcomes in these health markers at 18 months follow-up, with significant improvement in anxiety and depression scores [18]. Still, the significant distress that may be incurred by delaying the reconstruction process is a critical factor to consider during preoperative planning discussions, as damage to a patient’s psychosocial well-being is perhaps the biggest potential drawback to selecting a delayed approach. Ensuring a thorough understanding of this risk is essential to safeguard against incomplete disclosure and facilitate prophylactic mitigation strategies, such as providing access to mental health and social support services (Table 1).

This review aims to advance discussion at a particularly pivotal juncture in the evolution of breast reconstruction. The emergence of fascial-sparing, abdominally based flaps, the advancement of robotic and minimally invasive surgical techniques, and the increasingly sophisticated use of acellular dermal matrices (ADMs) and synthetic meshes mark a period of innovation in both immediate and delayed reconstruction. Such growth, however, has been accompanied by an evolving landscape of complication profiles that dictate shared decision-making conversations, inform risk prevention measures, and influence postoperative management. This review seeks to synthesize the current approaches to prevention, management, and treatment of delayed reconstructive complications to encourage patient-centered care, individualized operative planning, and improvement in overall outcomes.

### Delayed Breast Reconstruction Techniques

The reconstructive options available for delayed breast reconstruction mirror those used in immediate reconstruction and can be broadly categorized into three groups: autologous microsurgical reconstruction, implant-based reconstruction, and hybrid techniques. Each approach offers distinct advantages, limitations, and complication profiles, making them suitable or ill-adapted for certain patient populations. A thorough understanding of the risks and benefits of these options is essential for optimizing patient counseling and enhancing surgical outcomes in delayed breast reconstruction.

**Figure 1:**
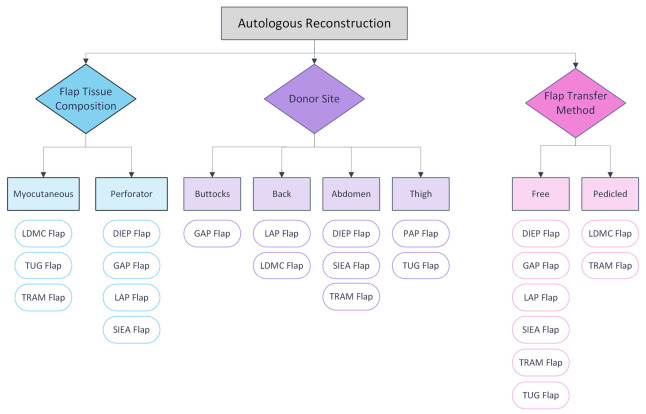
Techniques for Autologous Breast Reconstruction.

Autologous-based tissue reconstruction is most commonly performed using deep inferior epigastric perforator (DIEP) flaps, though alternatives may be employed if candidacy is limited by the abdominal fat pad, vascular anatomy, prior surgical history, or patient preference. Most options for breast reconstruction favor free tissue transfer, with key alternatives to the DIEP flap including the transverse upper gracilis (TUG) flap, transverse rectus abdominis myocutaneous (TRAM) flap, lumbar artery perforator (LAP) flap, or profunda artery perforator (PAP) flap [19]. Less common but viable flap choices are the latissimus dorsi myocutaneous (LDMC) flap, including the fat-augmented variant, which has seen great success in underweight patients, as well as gluteal artery perforator (GAP) or superficial inferior epigastric artery (SIEA) flaps [20,21] (Figure 1).

**Figure 2:**
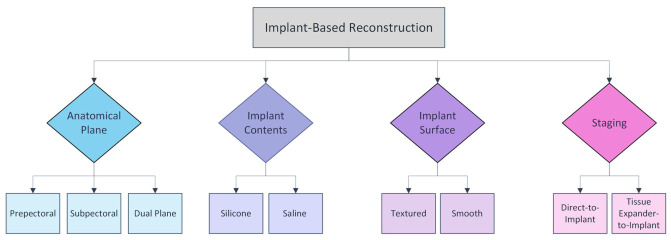
Techniques for Implant-Based Breast Reconstruction.

Although autologous-based reconstruction is often recommended given its association with higher patient satisfaction, factors such as patient anatomy, age or comorbidities, anticipated adjuvant therapy, surgeon or patient preference, and institutional resources may preclude microsurgical flap reconstruction. In such circumstances, implant-based reconstruction can deliver impressive aesthetic results through a shorter procedure and reduced recovery time, making it an appealing option for many patients. Implant-based reconstruction can be subdivided into two main categories: direct-to-implant approaches and a two-stage technique using tissue expanders to stretch the skin pocket before implant placement. Direct-to-implant reconstruction is generally preferred due to its shorter reconstructive course and immediate aesthetic results, although its feasibility may be limited by native breast anatomy or other patient factors. Two-stage implant-based reconstruction with tissue expanders offers an accessible and reliable alternative for women with limited or compromised overlying skin or a pocket that is incompatible with desired implant size, both of which are common concerns in post-mastectomy patients (Figure 2).

**Figure 3:**
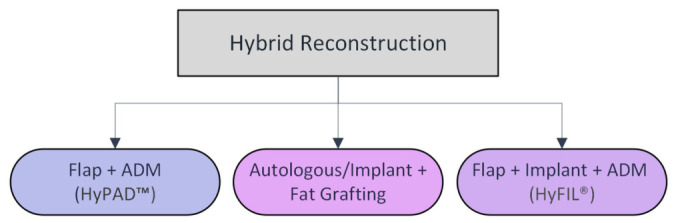
Techniques for Hybrid Breast Reconstruction.

Hybrid techniques are the latest innovation in breast reconstruction, combining the benefits of autologous reconstruction with greater core projection and volumetric enhancement than can be achieved with tissue-based reconstruction alone. For example, the HyFIL^®^ (Hybrid Flap, Implant, Lipofilling) technique involves placing a small implant between the autologous flap and chest wall, augmenting volume by roughly 120–250 ccs, depending on the implant used [22]. After securing the implant in the prepectoral space with an acellular dermal matrix (ADM) wrap, lipofilling may then be used to improve contour irregularities [23]. By contrast, the HyPAD™ (Hybrid Flap, Prepectoral Acellular Dermal Matrix) technique employs a folded ADM construct to provide an additional 70–140 ccs of breast volume [22]. Other hybrid modalities include the use of fat grafting combined with either purely autologous or implant-based reconstruction using standard-sized implant volumes rather than low-volume, mini-implants [24]. Technique selection may be volume-driven, with HyFIL^®^ offering the greatest opportunity for augmentation, or chosen based on other patient-specific factors such as contour irregularities (frequently fat grafting or HyFIL^®^) or breast pocket size incompatibility (for which HyPAD™ is often employed). Early evidence has not shown an association between the use of exogenous biomaterials such as ADMs and increased complication rates, supporting hybrid techniques as potential safe and effective strategies for improving aesthetic results among carefully selected patient populations [25]. Preliminary data has similarly demonstrated high success rates at an average of four years follow-up and after implant exchange, but given their relatively recent clinical implementation, such long-term outcomes studies remain limited and should be interpreted with caution [26,27] (Figure 3).

## 2. Methods

The authors partially conformed to published PRISMA (Preferred Reporting Items for Systematic Reviews and Meta-Analyses) guidelines and applied such processes to the following narrative review of complications related to delayed breast reconstruction procedures. Checklist items that were not relevant to the following report were either omitted or modified to accommodate the nature of this work.

The PubMed database was searched by two reviewers (L.C. and I.S.) for studies reporting data on complications related to implant-based, autologous, or hybrid breast reconstruction. Reference lists were scanned to search for additional sources of information related to the topic of interest. Studies were included based on outcome relevance; recently published articles from the last five years were preferentially selected for inclusion, though older foundational works were sometimes permitted if the findings were particularly notable or there existed few other comparable sources. Included sources were analyzed to confirm that the population of interest applied to reconstructive procedures rather than cosmetic surgery alone. Studies investigating complication rates of patients undergoing immediate reconstruction were not included unless delayed reconstruction patients were simultaneously studied.

## 3. Discussion

### 3.1. Complications of Delayed Autologous Breast Reconstruction

#### 3.1.1. Hematoma

An accumulation of blood in the breast pocket may result in hematoma formation, potentially compromising aesthetic results and, in severe cases, leading to ischemic pressure injury, hypotension, or concomitant infection. In a prospective study of 1212 autologous free flap breast reconstructions, immediate reconstructive approaches exhibited a hematoma rate of 7.4% compared to 5.2% for delayed reconstruction [28]. This difference likely reflects the increased procedural complexity of performing mastectomy and flap transfer concurrently, which results in prolonged operative times, greater vascular manipulation during anastomosis, and increased tissue trauma from immediate handling following excision. Moreover, the dead space created beneath the skin flap in immediate reconstruction may predispose patients to greater risk of blood accumulation relative to delayed procedures, in which skin pockets have been given time to heal. Still, hematomas may develop at other sites, such as the rectus sheath, at similar rates in either immediate or delayed autologous reconstructive techniques.

When hematomas do develop, management strategies include a prompt return to the operating room for evacuation with subsequent drain placement to prevent further fluid accumulation. In delayed reconstructive techniques, hematoma prevention can be achieved with judicious drain placement and monitoring, meticulous hemostasis, and intentional intraoperative blood pressure elevation to identify and contain bleeding from any low-pressure systems before closure. Extensive hemostasis must be ensured at both donor and recipient sites of microvascular dissection to prevent postoperative bleeding [28]. Risk factors for hematoma formation include the use of antiplatelet and anticoagulant medications, which should be discontinued prior to surgical intervention. Tranexamic acid (TXA) has demonstrated benefit in reducing hematoma risk and may be applied either intravenously or topically when judged appropriate for patient prophylaxis [29].

#### 3.1.2. Seroma

During mastectomy, lymphatic damage and inflammation resulting from tissue injury may encourage the production of proteinaceous and carbohydrate-rich fluid, resulting in seroma formation. In the context of delayed reconstruction, this initial period of tissue damage is given time to resolve, potentially conferring a lower baseline risk of seroma compared to immediate reconstruction [30,31]. Zolper et al. reinforce this notion, reporting that delayed autologous reconstruction without drain placement at the recipient site produced excellent results with a seroma rate of just 3.2% [30]. Another study reported a seroma rate that was nearly three times greater in the immediate group, a difference that retained significance even after adjustment for confounders [31]. Still, contention remains, and a 2021 systematic review and meta-analysis from Hershenhouse et al. found seroma and vessel thrombosis to be the only complications observed at significantly higher rates in delayed reconstruction compared with immediate timing (2.69% vs. 10.57%, *p* = 0.042) [32]. The use of tissue expanders was not accounted for, however, a caveat which may justify the higher-than-expected incidence [32].

Both immediate and delayed autologous techniques boast lower rates of seroma compared to implant-based approaches due to the absence of foreign material that may propagate an adverse inflammatory response. The same is true of purely autologous-based techniques compared with hybrid techniques that employ ADMs, as ADMs also carry potential to provoke an immunologic reaction. Moreover, breast reconstruction itself may reduce the risk of seroma development compared to isolated mastectomy, as reconstruction serves to either wholly or partially obliterate the dead space left by resection of native breast tissue [30]. Operative factors such as the extent of axillary lymph node dissection and number of nodes removed, or the use of electrocautery may influence the risk of seroma [33]. Demographic and postoperative care factors such as high BMI and elevated drain output may also increase the risk of seroma development [30,31,32,33]. Prevention strategies include the placement of surgical drains, flap fixation/quilting, shoulder immobilization, external compression, and the use of fibrin sealants [33]. Treatment strategies are often dependent on size and discomfort level, typically involving clinical observation or intervention with palpation- or ultrasound-guided aspiration [33]. Sclerosing agents may be utilized in a minority of cases to obliterate the seroma cavity, including tetracyclines, talc, saline, or iodine [33].

#### 3.1.3. Infection

A 2021 systematic review and meta-analysis reported higher rates of surgical site infection (SSI) in immediate breast reconstruction techniques compared to delayed methods, due in part to the ischemic environment created during mastectomy [32]. However, this difference did not achieve statistical significance (11.66% vs. 4.68%, *p* = 0.155). The overall literary consensus regarding the impact of reconstructive timing on infection risk favors the idea that infection rates are generally comparable between immediate and delayed approaches, approximating 7% overall [34,35]. While pTRAM and SIEA flaps have demonstrated higher rates of infection, no difference is observed in SSI incidence by flap type when postoperative antibiotic prophylaxis is given [34,36].

Infection often manifests as cellulitis, erythema, or abscess. These signs may be difficult to distinguish from normal peri-incisional erythema early on, especially when more blatant signs such as fever, pain, or drainage are absent. As a result, when infection is suspected, prompt investigation with cultures, laboratory tests, and ultrasound imaging should be initiated [33]. Patients undergoing delayed breast reconstruction may be particularly susceptible to infection when oncologic resection is combined with lymph node dissection. Other well-recognized risk factors include active smoking status, diabetes mellitus, use of steroids or other immunosuppressive agents, elevated body mass index, and mastectomy skin flap necrosis, while the influence of patient age remains inconsistently reported in the literature [33,34]. Antibiotic prophylaxis is recommended for all reconstructive techniques to help mitigate any opportunity for SSI: generally, antibiotics within 1 h of incision and discontinuation within 24 h postoperatively is sufficient [33,34].

#### 3.1.4. Wound Healing Difficulties

Wound healing complications are a primary concern in the context of delayed autologous breast reconstruction, often fueled by the joint effects of extensive tissue dissection, multiple operative sites, re-elevation of mastectomy skin flaps, and prior oncologic treatment. Though delayed reconstruction provides time for the skin envelope to heal, local tissues are often vascularly compromised from prior radiation therapy. As a result, complications such as skin necrosis, fat necrosis, and dehiscence may occur, reflecting either hypoxic tissue death (e.g., fat necrosis, skin necrosis) or structural breakdown (e.g., dehiscence). Overall wound complication rates for autologous reconstruction vary by flap type, with latissimus flaps demonstrating the lowest rates of wound complication (4.3%), followed by free flaps (6.2%), and pedicled TRAM flaps (8.1%) [37].

#### 3.1.5. Wound Dehiscence

The risk of wound separation, or dehiscence, is particularly elevated at the abdominal donor site, which is often closed under high tension. The incidence of wound dehiscence in delayed versus immediate reconstructions is variably reported in the literature, though, overall, minor wound complications appear not to be heavily influenced by reconstructive timing [32,34,38]. Notably, a frequently cited study from Momoh et al. found prior irradiation to be the only independent risk factor for wound dehiscence on logistic regression analysis, reflecting the potentially confounding influence of PMRT on complication profile, but this finding achieved only marginal significance (*p* = 0.049) [34]. Prevention strategies aim to reduce incisional tension through layered closure techniques or the use of abdominal binders. Treatment entails conservative measures focused on healing by secondary intention or placement of additional sutures to reestablish primary closure.

#### 3.1.6. Fat Necrosis

Fat necrosis is the most common recipient site complication, occurring in approximately 8.12% of delayed autologous reconstructions compared with 14.91% of immediate autologous reconstructions in the setting of PMRT [39]. Identifiable risk factors may include obesity, pre- or post-reconstruction radiation therapy, smoking, prior abdominal surgery, greater flap weight, and inadequate perforator caliber, number, or location [40,41,42]. Conversely, protective factors include supercharging (*p* < 0.001) and bilateral reconstruction (*p* = 0.01) [42]. A 2020 retrospective review of 866 free-flap breast reconstructions predicted that harvesting larger caliber perforators from the lateral row, either alone or in addition to medial row perforators, may help to decrease the rate of fat necrosis, though at the cost of increasing abdominal bulges [43]. Clinically, fat necrosis presents as a palpable mass or nodule in approximately 14% of cases, while ultrasound imaging detects a higher incidence, approaching 35% [39]. Smaller lesions (<1.97 cm) are more likely to resolve spontaneously than larger lesions (75% vs. only 35.3%, *p* = 0.041), with only 7% of cases requiring operative intervention [39,44].

#### 3.1.7. Skin Necrosis

Skin necrosis occurs nearly three times more frequently in autologous breast reconstruction compared with two-stage implant-based approaches, with reported rates of 30.4% versus 10.6%, respectively (*p* < 0.001) [45]. This marked disparity may be explained in part by the tendency for patients in the autologous cohort to have a concomitant diagnosis of diabetes or obesity, while also being significantly older and more likely to have had radiation therapy—all of which represent independent risk factors for skin flap necrosis, in addition to a history of tobacco use [45]. Interestingly, a prospective 2022 study published in *The Lancet* suggests that pre-operative radiotherapy may exhibit comparable rates of open breast wounds to post-mastectomy radiotherapy, though larger studies are needed to validate these results [46]. In a study by Tondu et al., rates of skin flap necrosis were 4.8% in single-stage vs. 1.43% in delayed approaches, suggesting that delayed reconstruction substantially reduces risk [47]. This higher incidence is likely multifactorial, driven in part by compromised perfusion of the mastectomy skin flaps after disruption of the native vascular network, as well as by lengthier surgical times in immediate approaches. Surgical technique also influences skin necrosis risk, with higher rates observed when circumferential peri-areolar incision lengths exceed 30% of the peri-areolar circumference, a greater breast tissue weight is excised, operative times are prolonged, or concurrent axillary dissection is performed through peri-areolar (22.4%) or radial (14.4%) incisions (*p* < 0.01) [48,49]. In autologous reconstruction, conservative methods of local wound care were often sufficient, succeeding in 37.1% of patients, while approximately 29% required operative intervention [45].

#### 3.1.8. Anastomotic Failure

Perhaps the most daunting complication of tissue-based reconstructions is the potential for microanastomotic failure and subsequent flap loss, resulting in significant reconstructive compromise. The overall incidence of flap failure remains low—variably reported between 2 and 6% depending on degree of failure and flap type—with most instances preceded by venous occlusion [50,51]. Pedicled flaps, such as the latissimus dorsi (LD) or TRAM flap, tend to experience lower rates of flap compromise compared with free flaps (<1% vs. 2–5%) due to the absence of a need for blood supply to be reconnected [51]. Arterial anastomotic compromise is significantly less common yet often more devastating due to the production of reactive oxygen species and a more critical degree of tissue hypoxia [52]. In instances of total flap loss (0.5–2.1%), the usual culprit is arterial in nature [50,51,53]. Methods of prevention center around adequate preoperative perforator selection and frequent intraoperative monitoring of perfusion status using Doppler ultrasound, indocyanine green SPY fluorescence, palpation, or an Acland strip test to ensure the rapid recognition of any changes in flap blood flow [54]. Imaging such as magnetic resonance (MR) or computed tomography (CT) angiography is almost universally used for perforator mapping in the preoperative setting, with 2–3 perforators originating from a mix of medial and lateral flap sites often recommended to maintain optimal perfusion requirements [54].

Since instances of flap loss require return to the operating room for flap removal, early detection of perfusion problems is essential to preventing the progression of flap ischemia and necrosis. If thrombosis is identified, systematic heparinization (5000 units) with mechanical thrombectomy and arterial administration of tissue plasminogen activator to the flap anastomosis may be performed [54]. Partial flap necrosis may be managed in some circumstances with hyperbaric oxygen therapy and conservative wound care, preserving flap vitality [55].

### 3.2. Complications of Delayed Implant-Based Reconstruction

#### 3.2.1. Hematoma

Delayed implant-based reconstruction demonstrates reduced hematoma risk compared to immediate reconstruction, approaching rates of 1–4% [56,57]. This is in line with previous literature suggesting a greater risk of complications in immediate reconstruction overall (unstratified by technique), with roughly 2-fold greater odds of developing a hematoma or seroma (OR 2.01, 95% CI 1.53–2.89) [58]. This difference is owed in part to decreased vascular manipulation and reduced dead space present when the procedure is delayed, while the completion of adjuvant chemotherapy may also permit platelet restoration. Indeed, pectoralis major is the primary source of bleeding in immediate reconstructions (50%), followed by axillary dissection [7,59]. The first stage of a two-stage expander-to-implant reconstruction carries a markedly higher risk of hematoma development, as noted by Lovecchio et al., who identified post-stage I radiation therapy as the only risk factor for complications in stage II (OR, 4.5; 95% CI, 1.4–15.2) [60].

Management strategies for hematoma in implant-based reconstruction parallel those used in autologous procedures, with return to the operating room for surgical evacuation remaining the primary therapeutic intervention. Several demographic factors may elevate hematoma risk, including the use of antithrombotic medications, older age, obesity, and smoking history [57,59,60]. Operative variables such as subpectoral versus prepectoral implant placement have not been shown to significantly predict complication rates, though individual surgeon and radiation therapy may influence risk [59,60,61]. Anticoagulant and antiplatelet agents should be limited or discontinued prior to surgery as a preventative measure. Notably, ketorolac has not been associated with increased hematoma incidence in implant-based reconstruction and may be an acceptable option for postoperative pain control [62].

#### 3.2.2. Seroma

Foreign implant materials may promote an inflammatory reaction in the surrounding tissue, leading to the production of serous exudate. The negative space created by the breast skin pocket provides a region for fluid accumulation to exceed lymphatic drainage, resulting in seroma. The overall incidence of seroma after mastectomy and implant-based reconstruction generally falls between 4 and 10% [56,63]. The effect of reconstructive timing on seroma rate in implant-based reconstruction is mixed: while a 2022 systematic review and meta-analysis found lower rates of seroma, one retrospective study on 138 patients found delayed reconstruction to be an independent risk factor for seroma development, though this finding barely achieved significance (*p* = 0.049) [58,64]. Factors such as smoking, obesity, and the use of synthetic mesh, larger native breast size, and prepectoral reconstruction may promote seroma formation [63,64]. Typical prevention measures entail the placement of pre and/or post-pectoral drains, which may be removed after output falls below 50 ccs for 2 consecutive days, ideally around 1–2 weeks postoperatively [63]. More novel prevention measures, such as Rifampin washes, have also been applied by some authors [63]. Treatment involves watchful waiting for natural drainage as well as clinical visits for percutaneous aspiration [63].

#### 3.2.3. Infection

SSI remains one of the most significant complications of implant-based breast reconstruction, given its potential to compromise reconstructive outcomes, precipitate sepsis, and necessitate removal of the implanted device. The inherent propensity of breast implant materials for bacterial adherence further increases the risk of infection with these procedures. In addition, implant-based reconstruction may impair perfusion at the mastectomy edges, resulting in hypoxia, skin necrosis, and delayed wound healing, conditions that further facilitate the colonization of bacteria. Reported infection rates for delayed implant-based reconstruction range approximately 3–6% compared to 8–9% in immediate, with studies demonstrating rates of 2.9% following initial tissue expander placement and approximately 4.1% after exchange to a permanent implant [8,58,65,66]. Immediate reconstruction is performed in a relatively hypovascular environment, as the implant is placed into a freshly created mastectomy pocket with disrupted skin flap perfusion. In contrast, delayed reconstruction occurs in well-vascularized, healed tissue, which may account for its comparatively lower infection risk. Importantly, a 2026 study by Paganini et al. found that operating time was independently associated with infection risk (OR 1.005 per minute, 95% CI 1.001–1.010; *p* = 0.010; corresponding to OR 1.38, 95% CI 1.08–1.77, per 60 min increment) [67]. Other risk factors specific to implant-based breast reconstruction include prepectoral implant placement, a history of tissue expander infection, the use of textured implants, axillary lymph node dissection, obesity, and post-mastectomy radiotherapy [65,67]. All infections should be treated with a course of oral or intravenous antibiotics, with severe or persistent symptoms alluding to the formation of a mature biofilm matrix that requires implant removal [67].

#### 3.2.4. Wound Healing Difficulties

Problems with wound healing or tissue compromise occur less frequently in implant-based reconstruction, likely due to decreased surgical site morbidity and less extensive dissection requirements. Fat necrosis is uncommon in the context of delayed implant-based breast reconstruction in the absence of fat grafting, as there is typically minimal autologous fat remaining in the mastectomy skin flap pocket to undergo necrosis. Similarly, data on wound dehiscence rates following delayed implant-based reconstruction is sparse. Olsen et al. report a combined rate of 4.1% for noninfectious wound healing complications—encompassing both fat necrosis and dehiscence—occurring within 90 days after delayed implant-based reconstruction [66]. Skin flap necrosis is also a relatively rare complication in delayed implant-based breast reconstruction, with one study observing zero instances of skin necrosis in the delayed cohort compared to 9.6% among the immediate reconstruction group (*p* = 0.004) [45]. These results are understandable given that delayed reconstruction is performed with well-vascularized skin envelopes as opposed to freshly raised mastectomy flaps in immediate reconstruction.

However, interpretation of these metrics is limited by the variability in the way skin necrosis is defined across studies. Many authors report wound dehiscence based on depth alone (ranging from superficial to full-thickness) or as a binary outcome (presence/absence) rather than severity [68,69,70]. In lieu of scoring systems such as the validated skin ischemia and necrosis (SKIN) scale, which only accounts for necrosis depth and area (with depth assessed by skin appearance and area approximated as a portion of the total breast), Oleck et al. propose a consensus definition that additionally incorporates intervention (e.g., debridement) and timing [70,71]. Overall, the current literature suggests that delayed implant reconstruction approaches are associated with lower rates of wound healing complications compared with immediate techniques, though standardized reporting metrics for wound dehiscence or skin necrosis would also be a welcome addition to the literature to improve cross-institutional variability.

#### 3.2.5. Capsular Contracture

**Figure 4:**
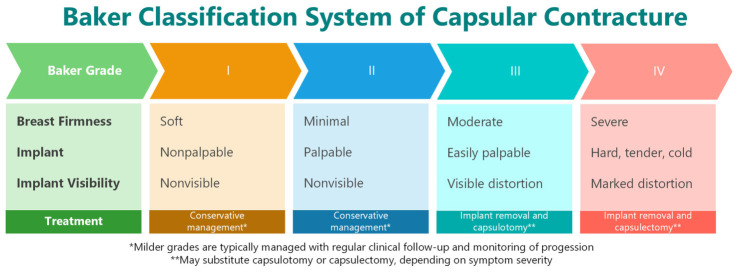
Baker Scale of Capsular Contracture.

Capsule formation represents a natural host response to foreign objects and, in the context of breast implants, effectively walls off the implant from the surrounding tissues. Though the capsule itself is typically benign and does not cause discomfort, capsular contracture represents a pathologic process involving progressive tightening of the implant capsule. Capsular contracture may occur in up to half of women with breast implants, producing chronic pain and aesthetic deformity [72]. The precise etiology of capsular contracture is unknown, though risk factors such as textured implants, implant rupture with leakage of silicone contents, infection leading to subsequent biofilm formation, prepectoral implant placement in the setting of radiotherapy, and previous capsular contracture or fibrosis have been shown to increase risk [72,73,74]. Importantly, PMRT may have a considerable impact on the likelihood of contracture [12]. In the absence of PMRT, capsular contracture rates around 30% are observed for delayed reconstructive techniques, while PMRT has been shown to produce Baker grade IV capsular contracture rates of up to 13.3% [10,75,76]. Whether irradiating a tissue expander or a permanent implant produces superior results remains to be elucidated, though popular belief favors tissue expander irradiation may provide some aesthetic benefit [10]. Iwahira et al. also contend that implant (OR 4.8 for 2 lateral fingers upward; OR 45.4 for 3 lateral fingers upward) or tissue expander (OR 3.4 for 1 lateral finger upward; OR 5.2 for 2 lateral fingers upward) misalignment heavily predicts capsular contracture rate using multivariable logistic regression analysis [75]. Treatment is heavily dictated by Baker classification type, ranging from conservative management to implant removal with complete capsulectomy. (Figure 4).

#### 3.2.6. Implant Malposition

A related complication that may occur in up to 32% of patients is implant malposition, which has numerous etiologies, including capsular contracture [77]. Implant malposition is a frequent occurrence in any implant-based reconstructive or cosmetic breast procedure, referring to the misalignment of the implant in the breast pocket. Textured implants were originally developed to combat this aesthetic concern but have since fallen out of use due to the association with BIA-ALCL. Clinical presentation of implant malposition varies widely, encompassing deformities such as pseudoptosis, double-bubble deformity, bottoming out, flipped implants, or a high-riding nipple, reflecting unidirectional, multidirectional, and rotational displacement categorizations [78]. Risk factors for implant displacement include larger implant volume (≥85 cc), smoking, and smooth round implants [79]. High BMI, dyslipidemia, and high projection implants have also been proposed as risk factors, though additional studies are needed to improve the reliability of these results [79].

Animation deformity is a specific type of dynamic implant malposition observed in cases of subpectoral implant placement. As the patient engages the pectoralis muscles, the posterior implant shifts underneath the newly contracted muscle, relocating to a position which affords more space. This new position is most frequently a superolateral displacement into the axilla, thereby producing an animation deformity. Subpectoral implant malposition can be corrected by repositioning the implants to the prepectoral plane, which limits the influence of muscle contraction on implant appearance. However, this may cause contour irregularity due to the loss of tissue coverage provided by the pectoralis muscle. In such cases, fat grafting and the use of ADMs may be utilized to improve the implant silhouette and reduce palpability [80].

Many other traditional instances of implant malposition may be fixed quickly in the clinical setting by performing maneuvers to redirect implant placement. However, some cases require a return to the operating room for permanent fixture, typically accomplished by suturing the implant to the neighboring breast parenchyma or chest wall to prevent unwanted movement. Implants may also be relocated to a different surgical plane, supported with a capsular flap, or secured with ADM [78].

#### 3.2.7. Implant Rupture

Implant leakage and rupture occur infrequently, with an estimated 5-year cumulative incidence of 3.1% for two-stage expander-to-implant reconstruction, though incidence appears to be intricately tied to implant type and follow-up period, reaching rates of over 30% for 10-year Mentor MemoryGel follow-up data [81,82]. Two kinds of fourth-generation implants—Allergan Biocell textured round and Allergan smooth round implants—have also been significantly associated with implant rupture compared to fifth-generation Mentor MemoryShape implants [81]. Other risk factors for rupture include older implants or longer indwelling time, and a shorter interval between staged operations (maximal discrimination observed at 6 months) [81,82,83].

Detection of implant rupture can often be confirmed with a combination of physical examination and diagnostic imaging [84]. Saline implant rupture is often easier to detect through physical examination alone compared to silicone implant rupture, as the absorption of saline filling into surrounding tissues creates a noticeable size discrepancy from the unruptured state. Rupture of silicone implants may be more occult, especially if intracapsular, potentially risking an immunologic reaction or rheumatologic process that eventually evokes detection [84,85]. Extracapsular silicone rupture behaves less discretely, presenting as a palpable mass, contour distortion, or granuloma [84]. Due to the difficulty of detecting rupture amongst silicone-filled prostheses, non-contrast MRI or ultrasound is generally recommended 5–6 years after silicone implant placement, with repeat screening every 2–3 years thereafter [84]. Management strategies for implant rupture in delayed breast reconstruction differ by implant type, with rupture of saline implants often addressed with expeditious replacement to preserve breast pocket size and rupture of silicone implants typically requiring capsulectomy and implant removal with or without implant replacement.

#### 3.2.8. Breast Implant-Associated Anaplastic Large Cell Lymphoma

Implant-associated cancer has been a topic of great interest in recent years, particularly the discovery of a malignancy that has almost exclusively been observed in patients with breast implants—breast implant-associated anaplastic large cell lymphoma (BIA-ALCL). Specifically, an association between textured breast implants and BIA-ALCL was established after a retrospective study revealed a 67.6 times greater likelihood of developing anaplastic large cell lymphoma in women with textured implants compared to the general population [86]. Cumulative data suggesting a strong causative link between exposure and diagnosis culminated in the Food and Drug Administration issuing a recall of textured implants in July of 2019. The precise etiology of this malignancy is unknown, but popular belief favors a chronic stimulation of the immune system by textured breast implants. Other theories suspect the etiology arises from a subclinical bacterial infection or genetic predisposition [86]. Most patients diagnosed with BIA-ALCL are in their mid-50s, typically 7–10 years post-implantation [86]. This diagnosis remains exceedingly rare in women without textured breast implants, though a high index of suspicion should be maintained in any individual experiencing delayed breast seroma, with aspiration and cytologic examination required for cautionary assessment of this clinical picture. Other less common presentations include a palpable mass (8–24% of cases) and palpable lymphadenopathy (4–12% of cases) [87]. As with other oncologic processes, treatment is largely dependent on staging but includes implant removal, complete capsulectomy, and resection of any associated tumor [67]. The rate of recurrence following surgical excision approximates 14% in patients with T4 disease compared with 0% in patients with T1-T3 disease; thus, the use of adjuvant chemotherapy or radiation is restricted only to those with advanced malignancy [87]. The incidence of BIA-ALCL remains low, with total lifetime risk estimated to fall between 1 in 1000 and 1 in 10,000 per year for women with textured breast prostheses [86].

A similar yet distinct kind of implant-derived malignancy is breast implant-associated squamous cell carcinoma, or BIA-SCC. Though presentations and preferred treatment methods for BIA-SCC largely parallel those of BIA-ALCL, the prevalence of this disease is exceedingly low, with only 19 cases and 3 deaths reported worldwide [88]. The disease course of BIA-SCC is comparably more aggressive, with higher rates of extracapsular invasion at the time of diagnosis as well as overall mortality [88].

#### 3.2.9. Breast Implant Illness

Social media has contributed to the notoriety of breast implant illness, or BII. BII is constituted by an array of non-descript symptoms such as rashes, malaise, fatigue, and muscle aches experienced by women who have received breast implants. As of yet, there is no definitive timeline for the onset of BII-associated symptoms, nor is the syndrome confirmed to originate from breast implants. The frequency of concomitant diagnoses in patients experiencing BII-like symptoms further complicates diagnosis [89]. Proponents of BII describe the disease as a collection of symptoms arising from an autoimmune-like phenomenon that some patients may experience in response to foreign implant material, and indeed many reported symptoms resemble those seen in conditions such as lupus, rheumatoid arthritis, and Hashimoto’s thyroiditis. A recent meta-analysis found high prevalences of psychiatric illness (16.5%), autoimmune conditions (20.7%), and fibromyalgia (12%) among women with BII symptoms, while implant rupture and contracture rates were 21.4% and 44.4%, respectively [90]. BII has also been reported as a social media phenomenon. Research demonstrates that 98.2% of BII patients seek online support and perform online searches of symptoms or treatment, suggesting that social media platforms may exacerbate anxiety and play into self-diagnosis [91]. BII remains a diagnosis of exclusion, and after other etiologies have been effectively ruled out, treatment includes implant removal. Further discussions may be required to develop alternative reconstructive approaches if desired by the patient.

### 3.3. Complications of Delayed Breast Reconstruction with Hybrid Techniques

Hybrid reconstruction techniques, including HyPAD™ or HyFIL^®^, integrate autologous tissue flaps with either ADM alone or ADM-wrapped small-volume implants. As a result, hybrid techniques are subject to a broad array of complications associated with autologous flap reconstruction, implant-based reconstruction, and ADM use. The use of smaller implant volumes in hybrid approaches may help mitigate certain risks affecting implant-only reconstruction, such as implant visibility, but cannot eliminate other risks inherent to implant use, such as BIA-ALCL and BII. Likewise, hybrid reconstructive modalities carry many of the risks associated with autologous reconstruction. Despite encompassing a complication profile representative of both techniques, hybrid techniques represent a favorable alternative to purely autologous or implant-based approaches for many patients. Desirable volume augmentation can be achieved or contour deformity corrected, while also maintaining a natural feel and appearance. These strengths make hybrid options ideal for patients in the delayed reconstructive setting for whom irradiation may pose significant functional and cosmetic threats, inducing fibrosis and contraction that neither autologous nor implant-based techniques alone may adequately address.

Combined breast reconstruction techniques generally demonstrate high success rates, often exceeding those observed in standalone approaches. In a 10-year single surgeon series of hybrid DIEP flap with implant reconstructions, success rates of 98% and 96.1% were achieved for initial implantation and subsequent exchange, respectively [27]. Similarly, Black et al. found that staged implant placement had a lower overall complication rate with a 97.5% implant/flap success rate, though revision was required in nearly half of all patients [92]. Between subcategorizations of hybrid modality, complication rate may differ significantly, as in the case of the fat-augmented LD flap in comparison to the LD flap with implant. While the LD with implant cohort experienced thirteen cases of capsular contracture and two extrusions, the fat-augmented group had only one instance of dehiscence and two oily cysts [93].

#### 3.3.1. Fat Necrosis

Fat necrosis is a common complication in autologous reconstruction but deserves mention in hybrid approaches, as the risk of tissue compromise is greatly increased with techniques such as lipofilling. In hybrid modalities such as HyFIL^®^ or standard-sized implant-based techniques with subsequent fat grafting, fat tissue is transferred without a source of perfusion, requiring successful integration into the recipient site for survival. Rates of fat necrosis therefore tend to be significantly higher in hybrid approaches with lipofilling or fat augmentation, approximating 10% compared with 2% of expander-to-implant exchanges without fat transfer [94]. Risk factors and treatment modalities mimic those seen in purely autologous reconstruction, with greater volumes of fat transfer raising the likelihood of necrosis.

#### 3.3.2. Red Breast Syndrome

Though the complication profiles of hybrid reconstruction techniques are largely a conglomerate of those seen in autologous and implant-based reconstruction, the use of ADMs introduces a unique risk that occurs in a minority of cases: red breast syndrome (RBS). Representing an inflammatory response after breast reconstruction with ADMs, RBS is characterized by erythema of the breast tissue that extends to the borders of the underlying ADM, with other symptoms such as swelling, pain, or edema typically absent except in severe manifestations [95]. The mechanism of RBS is incompletely understood, though several studies have postulated that a type I or IV sensitivity reaction directed towards the ADM or DNA remnants may be responsible [96,97]. As the exact mechanism of RBS remains elusive, prevention strategies are limited, though irrigating the ADM with saline and antibiotic solution prior to implantation has reportedly demonstrated some success [97]. Treatment typically consists of corticosteroids and antibiotics, though severe or refractory cases may necessitate ADM removal [97].

#### 3.3.3. Application to Clinical Practice

Techniques for breast reconstruction continue to evolve rapidly in sophistication, safety, and overall patient satisfaction. Reconstructive timing exerts a powerful effect on postoperative outcomes, influencing the risk of both long and short-term surgical complications, mental health, and recovery. During presurgical planning discussions, a comprehensive evaluation of the indications, complication profiles, risk factors, and management strategies unique to each reconstructive technique is an essential step to foster collaborative decision-making between an informed patient and transparent physician.

Unlike prior publications investigating this topic, this is the first literature review to discuss the unique complication profiles of all breast reconstruction categories. Overall, a critical evaluation of the literature reveals that the effect of timing on reconstructive outcome is intricately tied to the type of reconstruction being performed, inspiring the structure of this review to analyze technique-specific complication profiles. Implant-based reconstruction modalities exhibit the greatest disparity in timing-based complication rates, with delayed reconstruction boasting an odds ratio nearly 2-fold lower than that observed in immediate implant-based breast reconstruction [8]. By contrast, recent analyses on autologous reconstruction methods such as DIEP flaps have shown little variation in the rate of complications such as infection, hematoma, flap loss, or fat necrosis [38]. Between both groups, quality of life indicators do not differ significantly at long-term follow-ups approaching 2 years [5]. To date, there remains insufficient data on the timing of hybrid reconstruction to make definitive conclusions regarding whether immediate or delayed reconstructive methods achieve superior outcomes.

While preliminary evidence favors delayed reconstruction methods for implant-based approaches or as an indication for patients receiving PMRT, some limitations exclusive to delayed reconstruction do exist. Despite mitigating several adverse consequences of oncologic resection, delaying reconstruction also introduces the opportunity for extensive chest wall fibrosis to complicate reconstruction efforts. Likewise, post-radiation changes to the encasing breast pocket and surrounding tissue may pose threats to aesthetic results, including asymmetry, insufficient tissue laxity, and contracture (Table 2).

## 4. Conclusions

While less common than immediate reconstruction following mastectomy, delayed breast reconstruction may offer advantages for patients in need of post-mastectomy radiation therapy, or for those whose health status may not permit the physiologic stress of immediate reconstruction. Additionally, lower complication rates for delayed implant-based reconstruction confer optimal safety and reduce concern for a prolonged recovery course compared with immediate techniques. By providing an overarching review of the most common complications across delayed reconstructive modalities, a more comprehensive understanding of the steps needed to prevent and manage these potential obstacles to recovery may be achieved. A detailed knowledge of the risks involved, as well as strategies for risk mitigation, may facilitate shared decision-making while enhancing patient outcomes, safety, and satisfaction.

## Figures and Tables

**Table 1:** Pro/Con Comparison of Immediate and Delayed Breast Reconstruction.

	Immediate Breast Reconstruction	Delayed Breast Reconstruction
Advantages	Improved psychosocial well-being Lower cost	Possible reduced risk of immediate and long-term complications Lower VTE risk Potential expansion of patient candidacy (e.g., optimal for PMRT, high comorbidity burden, inflammatory breast cancer)
Pitfalls	Higher likelihood of revision surgery Extensive operating times Avoided in patients requiring PMRT	Poorer short-term outcomes in body image, sexuality, and psychosocial well-being Higher costs Longer total recovery time

**Table 2:** Indications, common complications, risk factors, and management strategies across breast reconstruction techniques.

	Considerations for Candidacy	Complications	Risk Factors	Management Strategies
**Autologous**	Younger age Presence of reliable donor site Amenable vascular anatomy Low comorbidity burden Support for recovery period Changes from PMRT making implant-based options impractical	Hematoma	Antiplatelets/anticoagulants	Operative drainage
		Seroma	Obesity, ALND *, and elevated drain output	Observation or aspiration
		Infection	Obesity, immunosuppression, diabetes, skin necrosis	Antibiotics with source control
		Dehiscence	PMRT, high-tension closure	Conservative management or reinforcement of primary closure
		Fat Necrosis	Obesity, smoking, PMRT, previous abdominal surgery, flap/perforator characteristics	Observation or operative removal
		Skin Necrosis	Obesity, smoking, PMRT, diabetes, operative variables	Conservative management, debridement, or skin grafting
**Implant**	Alignment with aesthetic goals (e.g., reconstruction with augmentation) Inability to tolerate longer operative/recovery time History of prior abdominal surgery precluding candidacy for autologous approach	Hematoma	Antiplatelets/anticoagulant	Operative drainage
		Seroma	Obesity, smoking, synthetic mesh, larger implant size, prepectoral placement	Observation or aspiration
		Infection	Obesity, history of implant infection, textured implants, ALND, prepectoral placement	Antibiotics, implant removal
		Capsular Contracture	Textured implants, implant rupture/infection/prepectoral placement, previous contracture, PMRT	Observation, implant removal/exchange with capsulectomy
		Implant Rupture	Older implants, implant type, shorter interval between staged reconstruction	Implant removal/exchange with/without capsulectomy
		Implant Malposition	High implant volume, smoking, smooth round implants	Physical maneuvers or operative revision
**Hybrid**	Insufficient volume from a single donor site Contour deformity Incompatible breast pocket size	Fat Necrosis	Autologous + high fat transfer volumes	Observation or operative removal
		Red Breast Syndrome	---	Steroids, antibiotics, or ADM removal

* Axillary lymph node dissection.

## Data Availability

No new data were created or analyzed in this study. Data sharing is not applicable to this article.

## Abbreviations

The following abbreviations are used in this manuscript:

HyFIL^®^	Hybrid Flap, Implant, Lipofilling
HyPAD™	Hybrid Flap, Prepectoral Acellular Dermal Matrix
